# Collimating individual beamlets in pencil beam scanning proton therapy, a dosimetric investigation

**DOI:** 10.3389/fonc.2022.1031340

**Published:** 2022-11-11

**Authors:** Jason Holmes, Jiajian Shen, Samir H. Patel, William W. Wong, Robert L. Foote, Martin Bues, Wei Liu

**Affiliations:** ^1^ Department of Radiation Oncology, Mayo Clinic, Phoenix, AZ, United States; ^2^ Department of Radiation Oncology, Mayo Clinic, Rochester, MN, United States

**Keywords:** lateral dose resolution, dynamic collimation, lateral penumbra, spot size, pencil beam scanning (PBS)

## Abstract

The purpose of this work is to investigate collimating individual proton beamlets from a dosimetric perspective and to introduce a new device concept, the spot scanning aperture (SSA). The SSA consists of a thin aperture with a small cylindrical opening attached to a robotics system, which allows the aperture to follow and align with individual beamlets during spot delivery. Additionally, a range shifter is incorporated (source-side) for treating shallow depths. Since the SSA trims beamlets spot by spot, the patient-facing portion of the device only needs to be large enough to trim a single proton beamlet. The SSA has been modelled in an open-source Monte-Carlo-based dose engine (MCsquare) to characterize its dosimetric properties in water at depths between 0 and 10 cm while varying the following parameters: the aperture material, thickness, distance to the water phantom, distance between the aperture and attached range shifter, and the aperture opening radius. Overall, the SSA greatly reduced spot sizes for all the aperture opening radii that were tested (1 – 4 mm), especially in comparison with the extended range shifter (ranger shifter placed at 30 cm from patient); greater than 50% when placed less than 10 cm away from the patient at depths in water less than 50 mm. The peak to entrance dose ratio and linear energy transfer was found to depend on the thickness of the aperture and therefore the aperture material. Neutron production rates were also investigated and discussed.

## 1 Introduction

A key technical milestone of proton therapy has been the advent and proliferation of pencil beam scanning (PBS) (1). PBS has made patient-specific apertures obsolete in many, but not all, scenarios, particularly for cases involving shallow and complex tumors (2, 3). The latest generation of proton machines typically have small in-air spot sizes, approximately 6 mm (σ at isocenter) at the lowest energies and 2 mm at the highest energies (4–14), yet because clinical accelerators cannot produce protons with energy lower than about 70 MeV, range shifters (2, 15–20) are required to treat shallow tumors. Unfortunately, range shifters, which may be 25 cm or more upstream of the patient, significantly increase spot sizes due to multiple Coulomb scattering in the range shifter and the subsequent divergence in space. The increased spot sizes reduce the overall lateral dose resolution, thereby resulting in poor protection of organs at risk (OAR) (21) that are in close proximity with shallow target volumes (8–10). This issue is especially significant in head and neck cancer treatment, where tumors are usually shallow and the number and proximity of OARs such as the salivary glands, spinal cord, brainstem, and optic-nerve structures are more pronounced (22–29).

In general, there is always a cost associated with increasing the lateral dose resolution. Each approach has advantages and disadvantages (30). One such approach is to use a patient-specific aperture. Unfortunately, the re-introduction of patient-specific apertures for PBS proton therapy (31, 32) counteracts one of the major motivations for PBS proton therapy, its adaptability. Moreover, patient-specific apertures, milled from brass to match the largest tumor cross-section from the beam’s eye view (plus some margin), only improve the lateral dose resolution at the depth with the largest tumor cross-section from the beam’s eye view.

Another aperture-based approach to improving the lateral dose resolution is to change the shape of the aperture dynamically during the treatment to conform to the tumor in the beam’s eye view for each energy layer. These apertures are known as dynamic apertures or dynamic collimators. One dynamic aperture has been made commercially available for Mevion proton machines, the so-called adaptive aperture (33, 34). The adaptive aperture is a multi-leaf collimator (MLC) developed for proton therapy, utilizing a relatively small number of thick nickel leafs. More traditional MLCs used in photon therapy (35, 36) have also been investigated for use in proton therapy (37, 38). However, for small tumors in proton therapy, MLC blades need to be thin (39) and yet still allow for a large field of view, a difficult challenge for MLCs. For this reason, the Mevion system limits the field of view to 20 cm x 20 cm. A recent and promising concept for dynamic collimation in proton therapy is the dynamic collimation system (40, 41) (DCS), consisting of four nickel bars (2 bars for trimming in x-dimension, 2 bars for trimming in y-dimension) that trim the fields by dynamically adjusting their positioning and orientation during the spot delivery. Like MLCs, the bars must be able to span the whole field of view. As a result, the treatable field of view using the DCS is currently limited to about 15 cm x 15 cm. While the adaptive aperture is available commercially for Mevion proton machines, the DCS is still under development. Both the adaptive aperture and the DCS use nickel as the aperture material in order to minimize the production of neutrons as compared to other high density metals (42).

**Figure 1:**
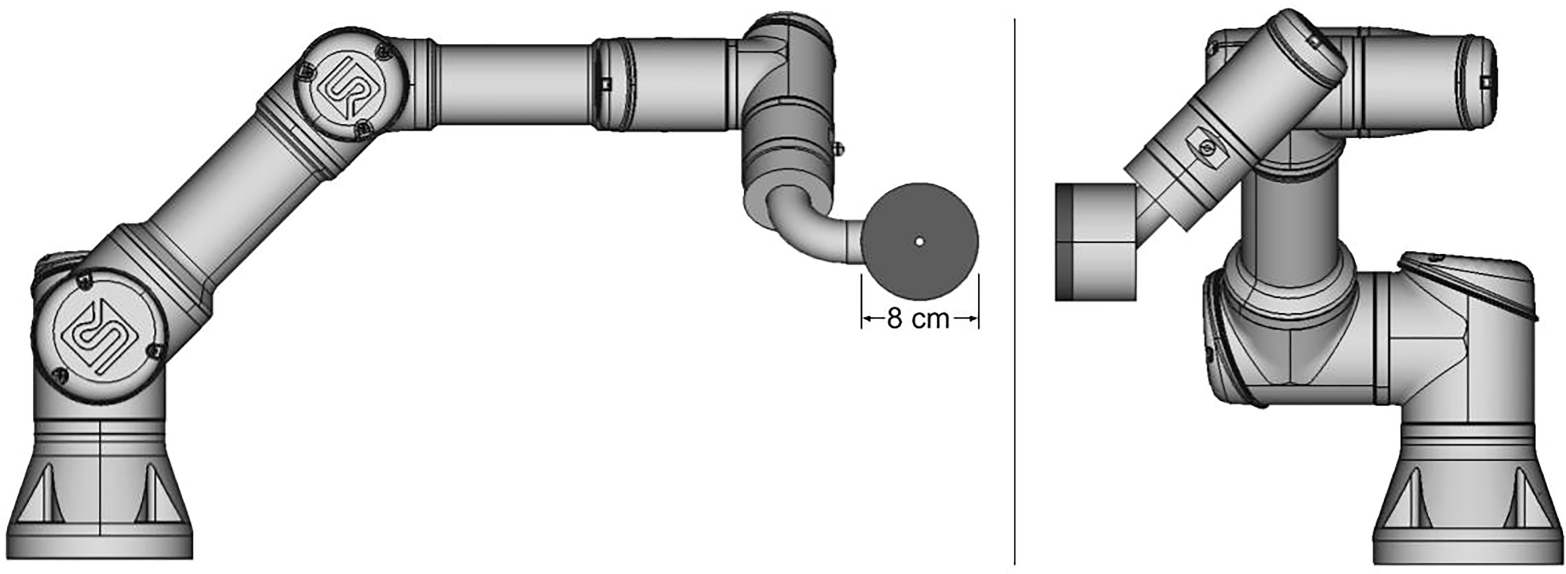
Diagram of a prototype spot scanning aperture (SSA) integrated with a collaborative robot, still under development. The aperture and range shifter assembly has a mass of about 2 kg.

In this work, we introduce a new aperture concept – an aperture designed to follow and trim individual beamlets spot by spot – a spot-scanning aperture (SSA) – rather than trimming individual field layers as in adaptive aperture and DCS. The SSA consists of three major components: a thin aperture with a single small cylindrical opening, a range shifter, and an advanced robotics system. A conceptual design for the SSA is depicted in **Figure 1**. Recent advances in robotics have led to a class of robots known as collaborative robots, which can move quickly with many degrees of freedom and with extreme repeatability well below the millimeter scale. The collaborative robot shown in **Figure 1** (Universal Robots UR3e, Universal Robots, Denmark) allows for motion in three-dimensions at up to 1 m/s with a reach of 500 mm while simultaneously aligning the cylindrical aperture opening to the beamlet axis with 0.03 mm repeatability. A large reach of 500 mm renders the device capable of covering a large field size in radiotherapy (as large as 40 cm x 40 cm commonly used in proton therapy). Since the SSA follows and aligns with individual beamlets, the aperture only needs to be large enough in the lateral dimension to block the outer extent of individual beamlets, which makes the patient-facing portion of the SSA (the aperture and range shifter assembly) much smaller than conventional apertures/collimators. The compactness and overall low mass of the patient-facing portion of the SSA, as depicted in **Figure 1**, should in principle allow for the aperture to be held close to the patient, an important aspect of any aperture designed for radiation therapy. Collaborative robots typically have many force sensors to prevent damage in an unforeseen collision.

Overall, the SSA concept aims to improve upon existing dynamic aperture designs with respect to field size, lateral resolution of individual beamlets throughout the tumor volume (not just at the edges), close placement to patients, and treatment adaptability. It should be noted that the full clinical implementation of the SSA is a systematic work that will require extensive efforts and teamwork between physicists, engineers, and proton vendors. This work is a preliminary feasibility study meant to illuminate advantages and weaknesses in the concept and to aid in the overall design prior to progressing towards a clinical device. Using an in-house customized open-source Monte-Carlo dose engine, MCsquare (2, 18, 43), we present an investigation into the dosimetric properties of the SSA.

## 2 Materials and methods

In order to investigate the dosimetric characteristics of the SSA, MCsquare was customized to include an aperture with a cylindrical hole, capable of being simulated with a range shifter. The open-source fast MC code, MCsquare (2, 17, 18, 20, 43) has been thoroughly validated against other MC codes and measurements in both phantoms and patient geometries (2, 18, 43–49). In addition, MCsquare has been fully commissioned and has been incorporated into our in-house treatment planning system, Shiva (6, 19, 50–55), and has been clinically used as the second monitor unit (MU) check system at our proton center for years (2, 18) and other proton centers (56). Therefore, we chose MCsquare in this study as a fast Monte Carlo dose and linear energy transfer (LET) calculation engine.

**Figure 2:**
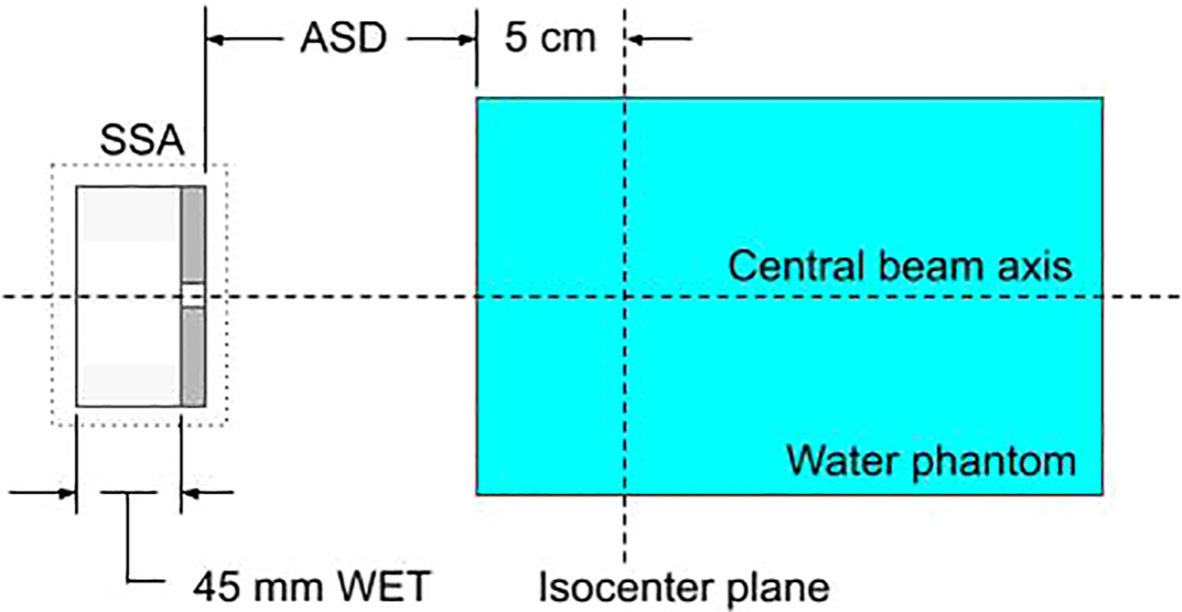
A diagram of the setup for characterizing the SSA within the Monte-Carlo simulation, MCsquare. Protons pass sequentially through the range shifter, the cylindrical opening of the SSA, and the water phantom. The figure shows a range shifter WET of 45 mm and zero gap between the range shifter and the aperture.

The SSA within MCsquare, as shown schematically in **Figure 2**, is defined by the following parameters: the range shifter water equivalent thickness (WET), the range shifter material, the range shifter to aperture distance (zero as shown in **Figure 2**), the aperture thickness, the aperture material, the aperture cylindrical hole radius, and the aperture to phantom surface distance (ASD). Because the collaborative robotic arm can align the cylindrical opening of the SSA with the beamlet axis no matter where the spot is located, the results for the beamlet passing through isocenter are assumed to be essentially identical to scenarios where the beamlet is aimed elsewhere. Therefore, hereafter the following studies in this work position the SSA such that the beamlet centroid passes through the center of the aperture opening and subsequently through the isocenter.

The approach for integrating the SSA into MCsquare was to mimic the pre-existing range shifter implementation, however using high density materials and changing the material within the cylindrical opening to air. During the dose calculation, protons reaching the aperture are tested at each step to determine whether they are within the cylindrical opening of the aperture or not. A proton is considered to be within the opening if its distance from the central axis of the cylindrical opening is less than the radius of the opening, else the proton is outside the opening. This method makes no approximation or simplification of the aperture opening. In a separate work investigating patient-specific apertures in PBS proton therapy (2), our customized version of MCsquare was found to accurately model apertures when compared to water measurements and RayStation (RaySearch Laboratories AB, Stockholm, Sweden).

### 2.1 Theoretical spot size limits

To establish a reference for spot sizes in water for which to compare with, we have simulated an infinitesimal proton beamlet normally incident on a water phantom (directed through isocenter) with 17 discrete energies ranging from 70 MeV to 230 MeV. The lateral spot sizes were obtained at the depth where the maximum dose was delivered, i.e., the Bragg peak (BP) depth. Since the beamlet begins with infinitesimal width and zero divergence, the resulting spot sizes represent the theoretical lower limit on spot sizes in water. For a more practical comparison, the spot sizes of the Hitachi system at Mayo Clinic Arizona were obtained through simulation without a range shifter (open beam) and with a 4.5 cm water-equivalent thickness (WET) extended range shifter (ERS) placed at 30 cm from isocenter.

### 2.2 Impact of SSA material, opening radius, and ASD upon spot sizes and integrated depth dose and LET distributions

Two SSA materials (tungsten and nickel), four aperture radii (1, 2, 3, 4 mm), and three ASDs (1, 50, 100 mm) were selected. Proton beamlets were simulated with nine energies (in-water Bragg peak depths ranging from 0 to 10 cm). Isocenter was chosen to be at 5 cm depth in the water phantom. Tungsten and nickel aperture physical thicknesses were 11.1 mm and 18.5 mm, respectively. These thicknesses were slightly greater than the minimum thickness required to stop protons capable of reaching depths of 10 cm in water. The simulation setup is shown in **Figure 2**. For each configuration, the spot size (σ) was obtained at the BP depth. For the case where the BP was at 5 cm depth, the 1D lateral profile as well as the integrated depth dose (IDD) and integrated dose-averaged LET (linear energy transfer) distribution (ILD) along the central axis was obtained with a voxel size of 1x1x1 mm (3). Additionally, to see how a moveable range shifter would compare to the SSA, a range shifter placed at the same distance as the SSA was simulated for each ASD.

### 2.3 Beamlet transmission

Another important consideration for the SSA to be used clinically is the beamlet transmission or the fraction of protons that pass through the aperture. The beamlet transmission was obtained by calculating the total dose for each SSA configuration divided by the total dose as calculated with no aperture given the same proton fluence. The beamlet transmission was obtained for tungsten and nickel apertures, with radii between 1-4 mm and a fixed ASD of 50 mm. Additionally, in order to measure the impact of spot positioning uncertainty on beamlet transmission, the beamlet was simulated with an offset error of 1 mm, an approximately two sigma error in spot position alignment to represent a worst-case misalignment of spot position for each scenario.

### 2.4 Impact of the distance between the range shifter and aperture on spot sizes and beamlet transmission

To study the impact of the distance between the range shifter and the aperture (within the SSA assembly) on spot sizes and beamlet transmission, we used a tungsten aperture with 3 mm opening radius at 50 mm fixed ASDs. The distance between the range shifter and aperture was increased from 0 mm to 300 mm along the central axis.

### 2.5 Relative neutron dose to a phantom as resulting from the use of different aperture materials

**Figure 3:**
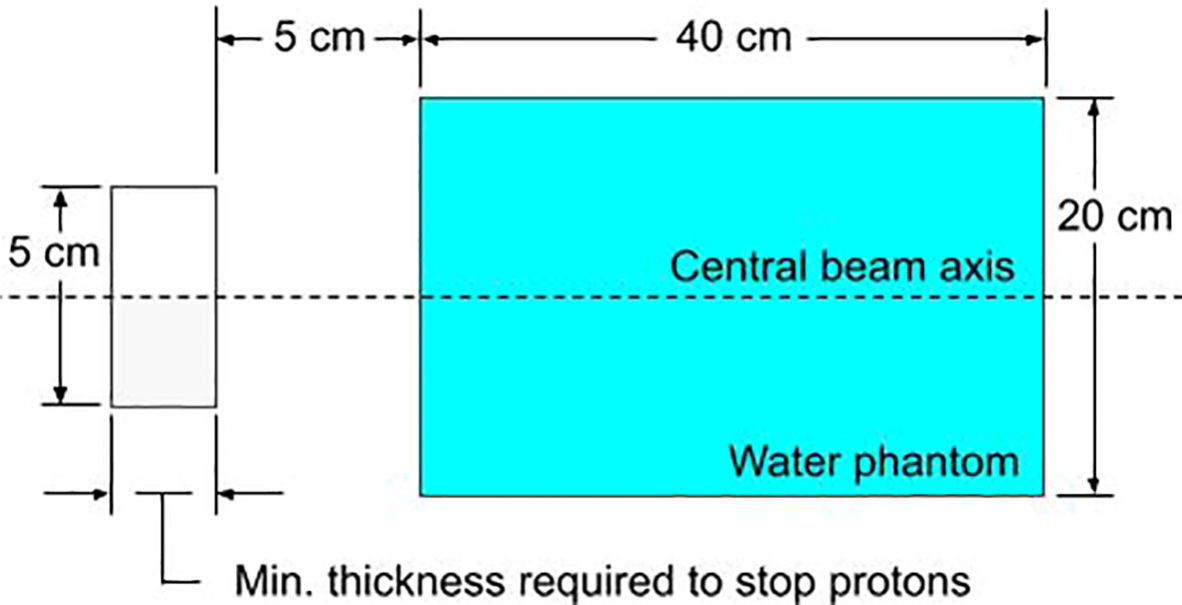
The simulated setup in TOPAS for evaluating the relative neutron dose for various block materials. The block aperture (gray) is cylindrical with a 5 cm diameter. The water phantom is cylindrical with a diameter of 20 cm.

Trimming individual beamlets will result in the production of neutrons during treatment. Since MCsquare does not simulate neutrons, neutron production was simulated using TOPAS (57) (version 3.7, reference physics list: QGSP_BIC_HP). An infinitesimal proton beamlet of 1E6 protons was directed towards a solid block of either tungsten, lead, brass, or nickel with the minimum thickness required to stop the protons (defined as the distance where the proton dose has reduced to one thousandth of the dose at the Bragg Peak plus 5%). **Figure 3** shows the simulation setup. The incident protons deposit all their energy into the solid block resulting in the production of neutrons, some of which deposit dose into the downstream water phantom. This substudy closely resembles the study performed by Gustafsson et. al (42)., where the same setup was simulated in Monte Carlo N-Particle eXtended (MCNPX). The context of their study was passive scattering, only simulating 230 MeV protons. Since this work is focused on depths up to 10 cm using PBS, we wanted to compare relative production rates of neutrons for apertures with a maximum treatment depth of 10 cm. However, in order to compare our results with Gustafsson et. al, we simulated up to 230 MeV.

### 2.6 Integral neutron dose estimates for H&N patients

The excess integral neutron dose as contributed by the SSA was estimated for 45 H&N proton PBS treatment plans that had been delivered previously. The integral neutron dose was estimated on a per-field basis for fields where the treatment depth minimum was less than 4 cm (water-equivalent) and the treatment depth maximum was less than 10 cm depth. The dose estimate is calculated for each spot by first determining the number of protons that will be absorbed by the aperture (based on the beamlet transmission results, Section 2.3), then determining the neutron dose based on the number of protons absorbed (based on the neutron dose results, Section 2.5). The total number of protons used in the treatment as well as the number of protons absorbed must be increased based on the percentage of protons that are absorbed such that the number of protons passing through the aperture is the same that was originally planned without the SSA.

## 3 Results

### 3.1 Theoretical spot size limits

**Figure 4:**
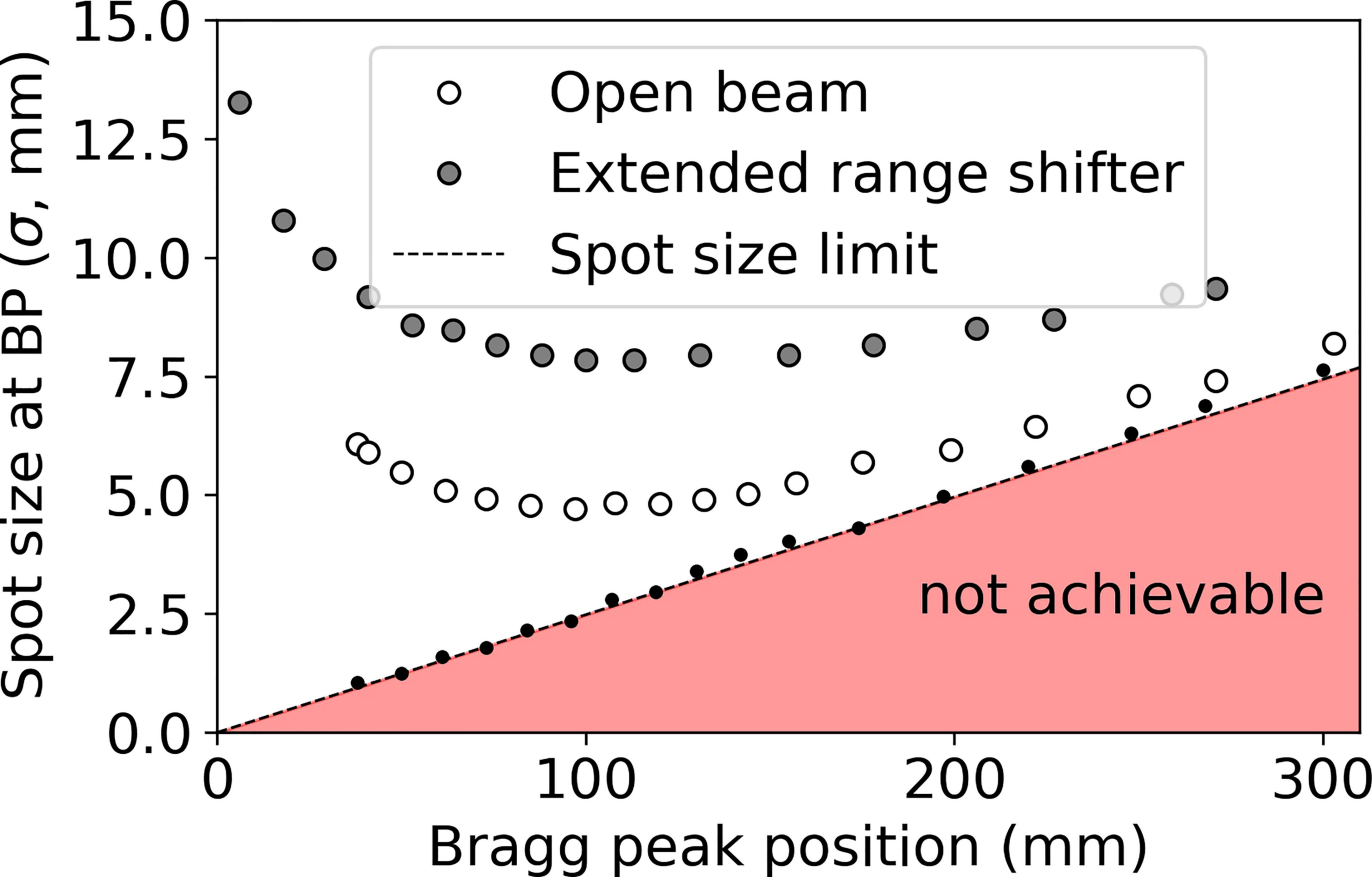
Spot sizes (σ) obtained in water under three conditions: infinitesimal beamlet (black dots, dotted line), open beam (no beam-modification devices beyond the exit of the nozzle, white dots with black outline), and including a 45 mm WET range shifter at the extended position 30 cm upstream of isocenter (ERS, gray dots with black outline).

**Figure 4** shows the theoretical lower limit on spot sizes in water (black dots), practical spot sizes under conditions of open beam (white dots with black outline) and ERS (large gray dots with black outline), obtained in water at the BP depth. **Figure 4** shows that, beyond 15 cm water-equivalent depth, there is little opportunity for reducing spot sizes further since the open beam is approaching the theoretical limit. The greatest opportunity for reducing spot sizes at Mayo Clinic Arizona is at depths of less than 10 cm, especially at depths less than 4 cm where the use of a range shifter is required. By reducing spot sizes at these shallow depths, the dose conformity to the tumor (58) may be greatly improved.

### 3.2 Impact of SSA material, opening radius, and ASD upon spot sizes and integrated depth dose and LET distributions

**Figure 5:**
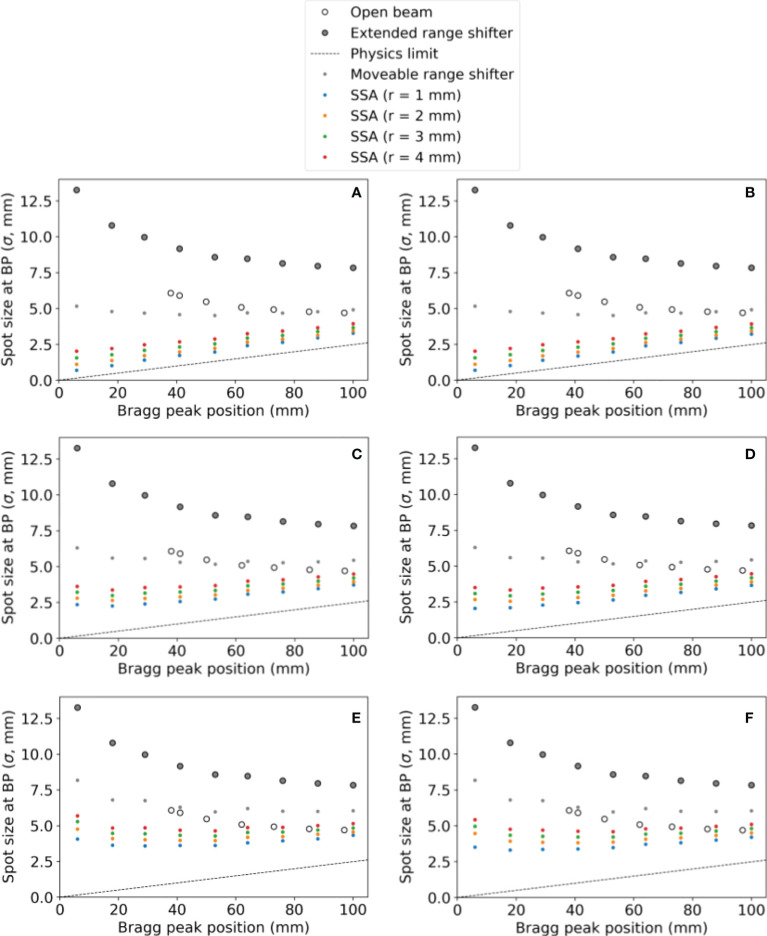
The spot sizes at the BP position for different SSA configurations (aperture opening radius and ASDs), for the extended range shifter (ERS), for the open beam, and for a moveable range shifter. Tungsten results are on the left and nickel results are on the right. The aperture distance to the water phantom surface was **(A, B)** 1 mm, **(C, D)** 50 mm, **(E, F)** 100 mm. The radius of the SSA varied from 1 mm to 4 mm as indicated by different colors in the figure.

Varying the proton energy and the ASD, the resulting spot sizes are shown in **Figure 5** with the ASD increasing from the top to bottom as follows: *(a)(b)* 1 mm, *(c)(d)* 50 mm, and *(e)(f)* 100 mm. In **Figure 5**, the tungsten results are on the left and the nickel results are on the right. The SSA results are plotted alongside the results from “Open beam”, “Extended range shifter” and “Moveable range shifter”. The results for tungsten and nickel are very similar, however with nickel having slightly smaller spot sizes due to its greater thickness and hence greater collimation power. Regardless of the material/thickness, the SSA significantly reduces spot sizes at depths where a range shifter is required (depths less than 4 cm) compared to the ERS or the moveable range shifter placed at the same distance to the phantom as the SSA. As expected, the greatest reduction in spot sizes occur when the SSA is positioned against the phantom surface, i.e., ASD = 1 mm.

**Figure 6:**
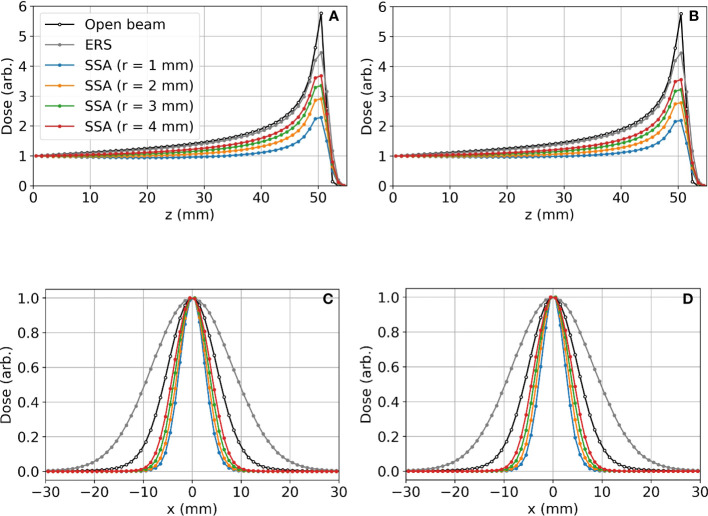
Lateral proton spot dose and IDD profiles for different SSA configurations where the BP is about 50 mm deep in the water phantom: **(A)** IDD profiles for tungsten; **(B)** IDD profiles for nickel; **(C)** Transverse proton spot dose profiles at the BP depth for tungsten; **(D)** Transverse proton spot dose profiles at the BP depth for nickel. For these data, the ASD was 50 mm. The radius (*r*) of the SSA opening for both materials was varied from 1 mm to 4 mm indicated by different colors in the figure.

**Figure 7:**
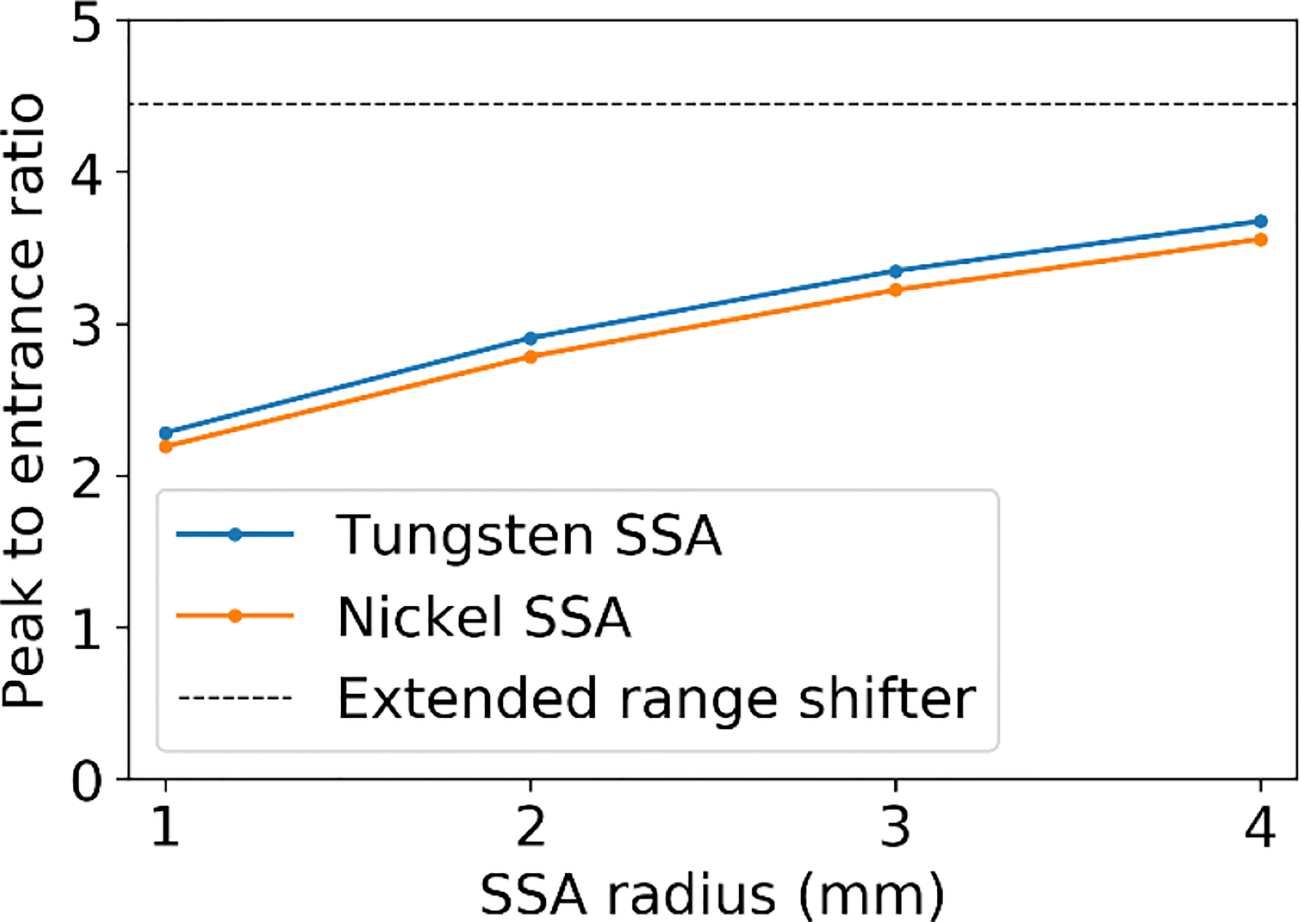
The peak to entrance dose ratio for the SSA with aperture opening radii varying from 1 to 4 mm and an ASD of 50 mm.

**Figure 6** shows (*a*)*(b)* the IDD profiles and (*c*)*(d)* the transverse profiles of the proton spot dose at the BP depth with the tungsten results on the left and nickel results on the right. The IDDs shown in **Figure 6** are normalized such that the entrance dose for each is set to 1. This normalization allows for visualizing the effect of the aperture radii on the shape of the IDD curves. As the radius of the SSA opening is increased, the IDDs gradually approaches that of the extended range shifter (*gray* lines) since the SSA also contains a range shifter. The results for both aperture materials are similar, however, the thinner tungsten aperture (11.1 mm) has a slightly higher peak to entrance dose ratio than the thicker nickel counterpart (18.5 mm). For either material, there is a tradeoff between the peak to entrance dose ratio and smaller spot sizes. The peak to entrance dose ratio is plotted in **Figure 7**.

**Figure 8:**
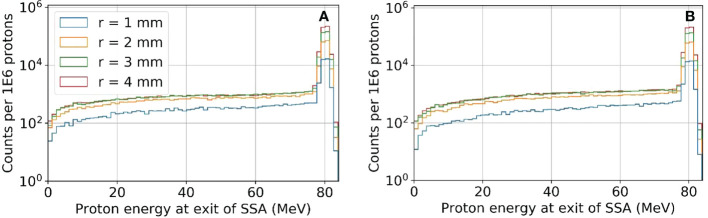
The proton energy distribution at the exit of the SSA for tungsten **(A)** and nickel **(B)** where the ASD was 50 mm and a primary energy of about 80 MeV and BP of about 50 mm.

**Figure 9:**
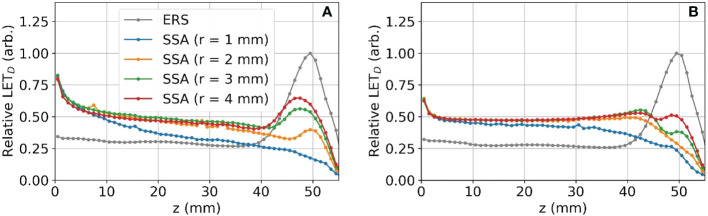
The dose-averaged LET integrated along the z-axis for tungsten **(A)** and nickel **(B)** SSAs.

The reduction in the peak to entrance dose ratio is caused by a non-negligible percentage of protons that interact with the aperture before reaching the phantom. These secondary protons reach the phantom with lower energy depositing energy at depths proximal of the primary proton BP. The energy distributions for the protons exiting the aperture (same setup as **Figures 6** and **7**) are shown in **Figure 8**. As a result of the wide energy distribution of the secondary protons exiting the aperture, the beamlet LET is also affected. The dose-averaged LET integrated along the central beam axis is shown in **Figure 9**. The LET varies significantly between the tungsten and nickel aperture. The tungsten aperture creates a larger LET at the entrance and is more peaked approaching the BP while the nickel aperture causes a flatter LET overall.

### 3.3 Beamlet transmission

**Figure 10:**
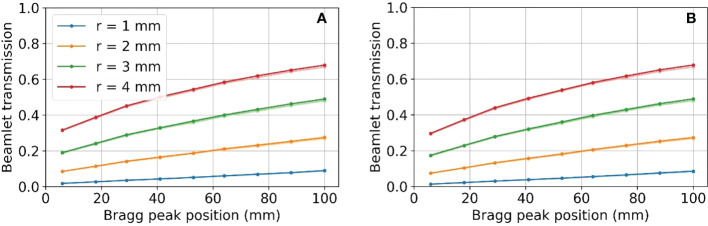
Beamlet transmission for SSA opening radii from 1 to 4 mm, placed at an ASD of 50 mm for **(A)** tungsten on the left and **(B)** nickel on the right. Lighter colored lines represent the resulting beamlet transmission where there was a 1 mm misalignment of the aperture. Two lines are almost overlapped with each other.

**Figure 10** shows the resulting beamlet transmission ratios for tungsten (left) and nickel (right) when the radius of the aperture opening is varied from 1 to 4 mm. Although not shown, the beamlet transmission varies slightly as the SSA is moved to/away from the water phantom, however the dominant parameter affecting the beamlet transmission is the aperture radius. The tungsten apertures allow for slightly higher beamlet transmission as compared to nickel. The beamlet transmission is the smallest at shallow depths, to be expected since the lowest proton energies have the largest spot sizes and are more heavily trimmed by the aperture. As **Figure 10** shows, the beamlet transmission spans a large range from 0.01 to about 0.7 depending on the SSA opening radius and the BP depth. The effect of misalignment between the aperture position and the spot position by 1 mm, an approximately two sigma error in spot position alignment, was to reduce the beamlet transmission slightly as shown in **Figure 10** with lighter colored lines. The impact of this worst-case spot position misalignment is hardly visible (two lines are almost overlapped with each other) as a result of the range shifter in the SSA scattering the proton phase space prior to collimation. The exact positioning of the aperture relative to the spot position is unimportant so long as misalignments are less than 1 mm. Based on this result, robotics repeatability of less than one millimeter, as specified by most collaborative robots, is adequate.

### 3.4 Impact of the distance between the range shifter and aperture on spot sizes and beamlet transmission

**Figure 11:**
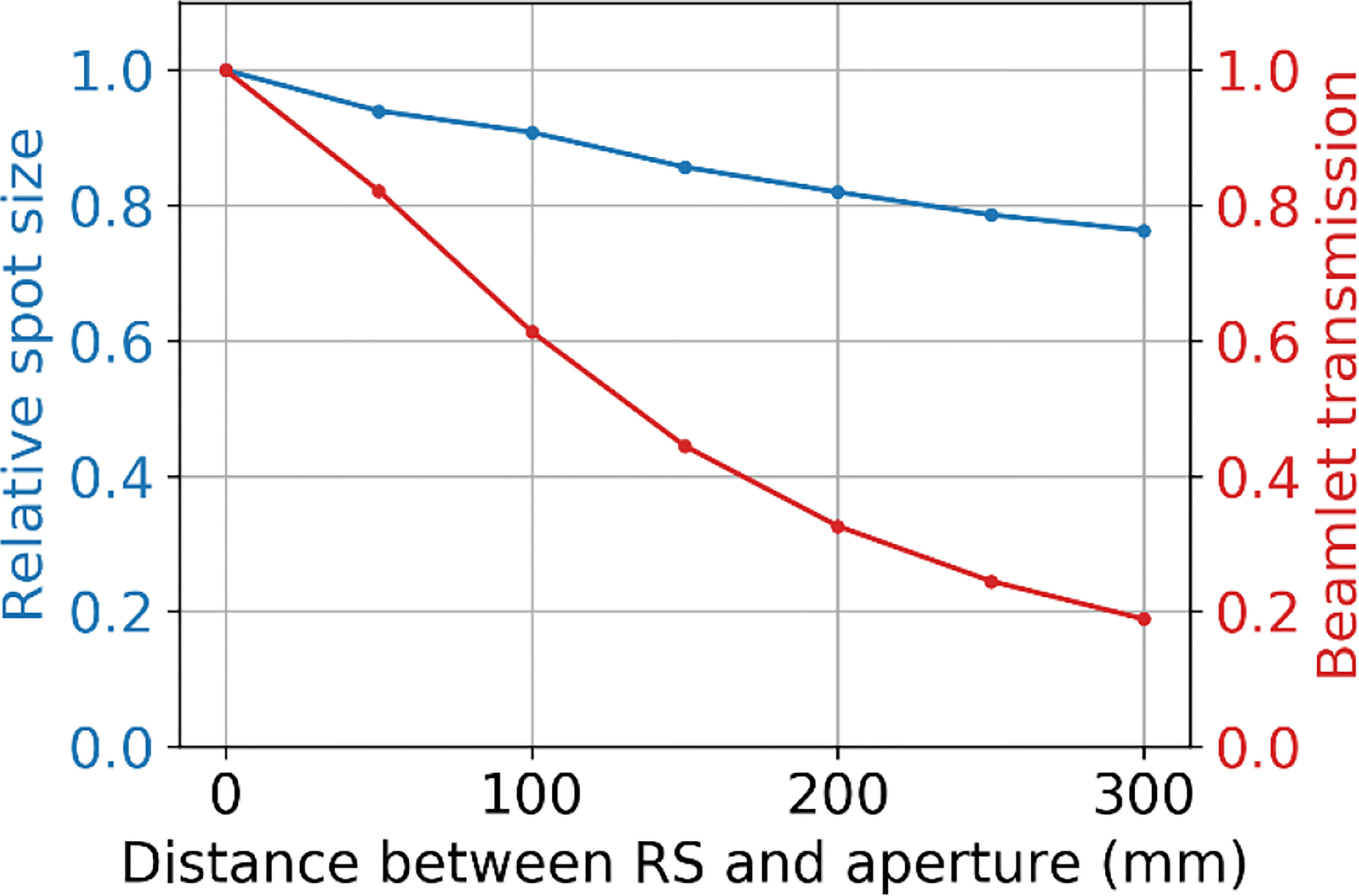
The relative spot size at the BP depth (50 mm) and beamlet transmission as a function of the separation distance between the range shifter and the aperture, where the tungsten aperture with a 3 mm radius aperture opening is at 50 mm from the water phantom surface.

**Figure 11** shows the relative effect of increasing the distance between the range shifter and aperture on the spot size (at the BP) and the beamlet transmission. These results show that as the distance between the range shifter and aperture is increased, the beamlet transmission reduces at about four times the rate that the spot size reduces.

### 3.5 Relative neutron dose to a phantom as resulting from the use of different aperture materials

**Figure 12:**
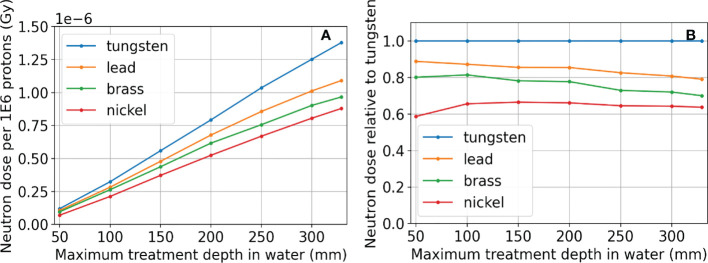
Neutron dose results for tungsten, lead, brass, and nickel, where the proton energy was the maximum possible for each aperture thickness. Absolute dose results are in **(A)**. The neutron doses resulting from apertures using different materials relative to using tungsten are shown in **(B)**. The aperture thickness is 5% thicker than the distance where proton dose reduces to one thousandth of the dose after the Bragg peak in water (Maximum treatment depth in water) and therefore depends on the aperture material. The maximum proton energy simulated was 230 MeV.

The neutron dose results are shown in **Figure 12**. The total dose resulting from neutrons is considerably higher at deep depths than at shallow depths regardless of the aperture material. At all treatment depths, the dose resulting from neutrons for the nickel aperture was between 60%-70% that of the dose resulting from a tungsten aperture as shown in **Figure 12B**. This result is consistent with Gustafsson et. al (42). where the ratio of equivalent dose in terms of Sieverts was 70% for 230 MeV protons. The results were also consistent for lead and brass. In the figure, maximum treatment depth in water is defined to be the distance where proton dose reduces to one thousandth of the dose after the Bragg peak in water. The aperture thickness is 5% thicker than the maximum treatment depth in water and therefore depends on the aperture material. The proton energy was the maximum possible for each aperture thickness. The maximum proton energy simulated was 230 MeV.

### 3.6 Integral neutron dose estimates for head and neck patients

**Figure 13:**
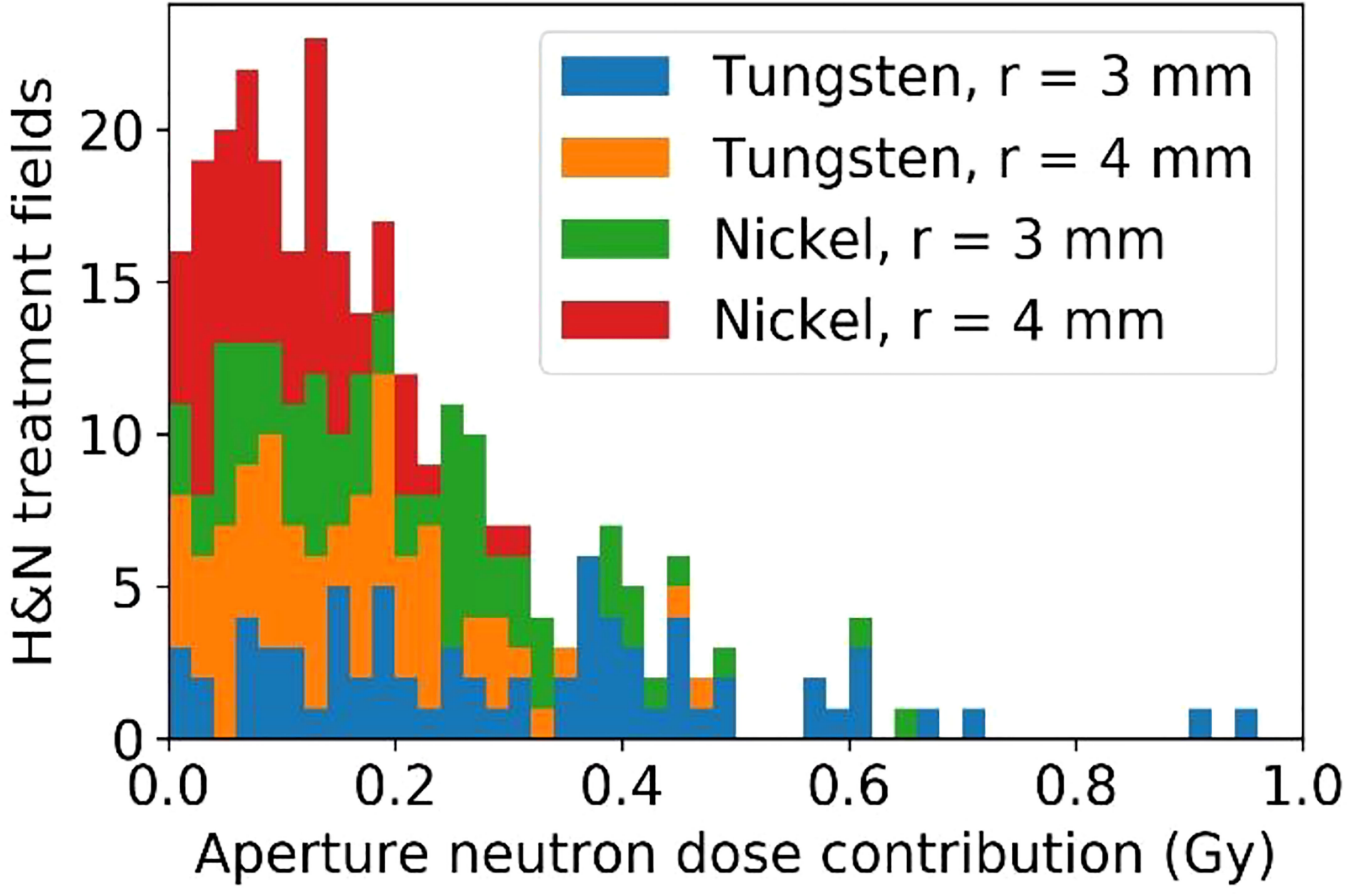
The estimated excess integral neutron dose as produced by the tungsten or nickel aperture in the SSA for 45 H&N treatment plans that were delivered previously.

Of the 45 H&N patients selected for integral neutron dose estimates, 72 fields were selected for analysis having met the restriction of the treatment depth starting at depths less than 4 cm and extending to no deeper than 10 cm. These per-field results are presented in terms of physical dose (Gy) to avoid potential bias since in this case, the neutron source (aperture) is close to the patient and will not produce a whole-body dose. Even still, it is likely that the effective dose (photon-equivalent dose) will be higher by perhaps a factor of 10 or more. From **Figure 13**, the per-field integral neutron dose is fairly high regardless of the configuration. The mean integral neutron dose was 0.30 Gy, 0.15 Gy, 0.21 Gy, and 0.10 Gy for the tungsten aperture with 3 mm radius, tungsten aperture with 4 mm radius, nickel aperture with 3 mm radius, and nickel aperture with 4 mm radius respectively. This indicates that changing the radius by 1 mm is more significant than the choice in material as the tungsten aperture with 4 mm radius was estimated to produce less integral neutron dose than the nickel aperture with a 3 mm radius.

## 4 Discussion

In this work, we have explored the concept of trimming individual beamlets in order to improve lateral dose resolution for shallow tumors, the spot scanning aperture or SSA. The concept is simple and yet unexplored in the literature, most likely due to the challenging robotics requirements. With the emergence of highly advanced collaborative robots, which can move at speeds of up to 1 m/s with submillimeter repeatability, trimming individual beamlets is now possible. The question then becomes whether it makes sense to collimate individual beamlets in the first place. This study begins to answer that question by studying the dosimetry of the SSA. While the ability to reduce spot sizes with the SSA was never really in question, we wanted to examine the quality of its dosimetry over a wide parameter space. In other words, can the spot sizes be reduced significantly using the SSA while retaining dosimetric characteristics resembling the familiar proton dose depth curve?

The proton therapy facility at Mayo Clinic Arizona has relatively small spot sizes when compared to most facilities. The results of this study are therefore a conservative representation of the benefits that could be offered by the SSA. Facilities with much larger spot sizes would be expected to have a greater benefit in terms of spot size reduction, albeit with unique dosimetric characteristics that will constrain the design. While the exact dosimetric benefits of the SSA will vary from facility to facility, the results in this work can be used for general understanding as well as a template for tailoring the design.

Based on the results, we can begin to formulate design constraints and possible clinical applications for the SSA. The 1 mm radius aperture is practically unusable due to its very low beamlet transmission ranging between 1-10% as well as its poor peak dose to entrance dose ratio, about 50% of the one with ERS. The 2 mm radius may be usable for small tumors close to the surface. For ocular tumors, which are very shallow and small, the 2 mm radius has ideal dosimetric properties, however the beamlet transmission ranges between about 10-30%, which would require somewhere between 3-10 times the overall spot fluence as the one with no aperture. Depending on the dose rate capabilities of the facility, this may be a challenge. One other beneficial aspect of treating ocular cancers with the SSA is that the patient facing portion of the SSA could be placed very close to the patient, resulting in spot sizes (σ) of about 1-2 mm (see **Figure 5**), possibly small enough to allow for treating ocular tumors with PBS (59). A 3 mm radius gives a beamlet transmission between about 20-50%, therefore requiring between about 2-5 times the overall spot fluence as the one with no aperture, softening the dose rate requirements (**Figure 5**). Finally, since the integral neutron dose depends heavily on the treatment depth, the risk for secondary cancers may be reduced to safe levels for very shallow tumors.

**Figure 14:**
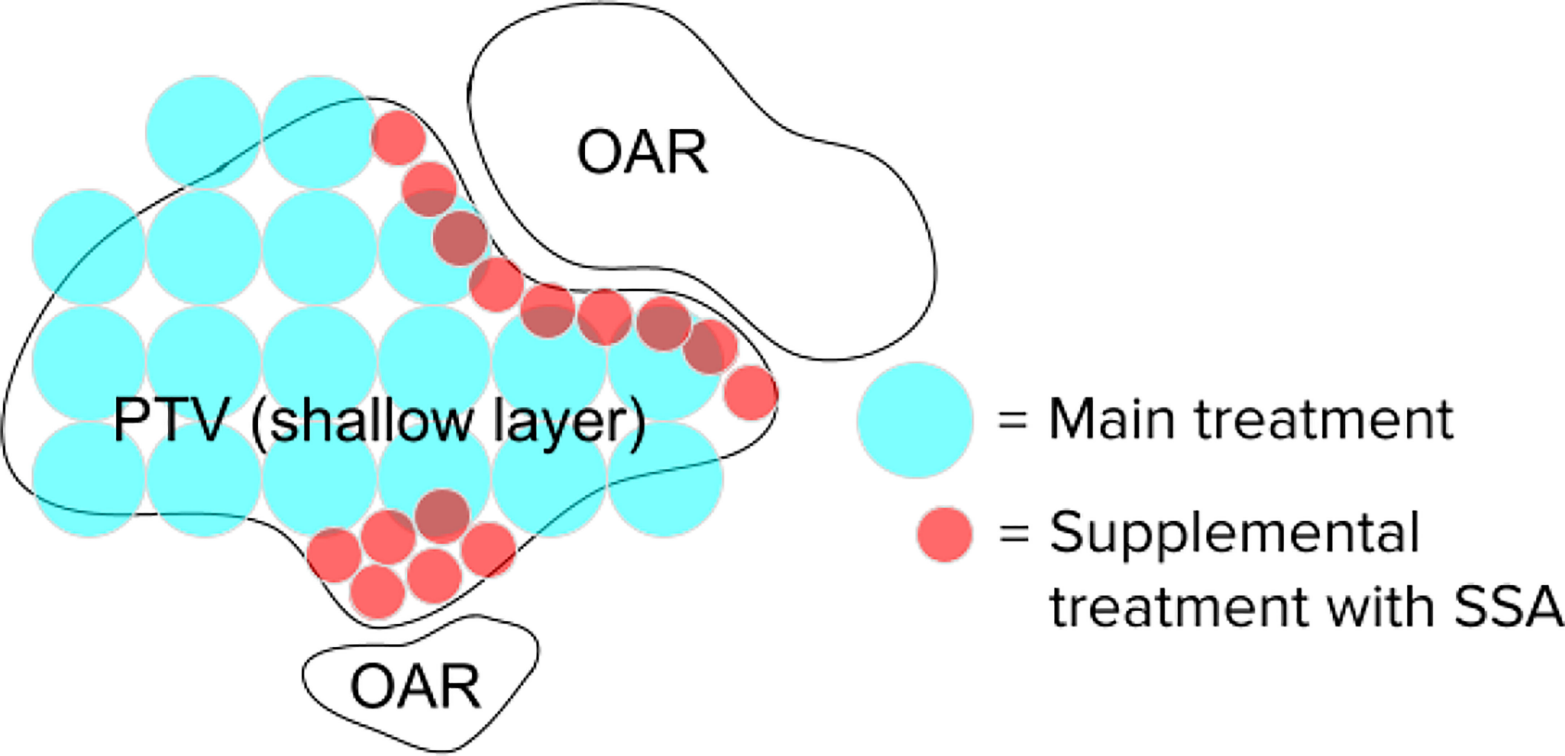
Hypothetical spot positions for a single layer, for the main treatment using conventional techniques and the supplemental spots using the SSA. The SSA could be moved out of place when not needed. This will significantly reduce the beam-on time and excess integral neutron dose.

The 3 mm and 4 mm aperture opening radius SSAs resulted in good overall dosimetry over the many SSA parameters that were evaluated, albeit with somewhat poor LET characteristics and somewhat high estimated integral neutron dose in the H&N patients. The 3 mm and 4 mm radius gave about 75% and 85% peak dose to entrance dose ratio respectively, relative to the one with ERS. At treatment depths of less than 5 cm, the SSA spot sizes are significantly smaller than either the range shifter placed at the same position as SSA, the open beam, or the ERS. This is important since in clinical practice a range shifter must be used to treat these shallow depth tumors. At depths between 5 cm and 10 cm, the spot sizes are still smaller, however the distance between the patient and the aperture becomes more important. If the aperture is placed at greater than about 10 cm from the patient, then the device should be used only for very shallow tumors. Based on these dosimetric characteristics, the 3 mm and 4 mm radius SSA may be well suited for complex and shallow H&N cancers. For a tumor that extends from a shallow depth to beyond 10 cm depth, the SSA could be moved out of place for deep layers. Similarly, to avoid excess integral neutron dose and high entrance LET overall as well as additional treatment time, the SSA seems best suited to irradiate around the boundary of tumor volumes, or near organs at risk, as a supplemental treatment, as demonstrated in **Figure 14**.

The proximity of the SSA to the patient is clearly an important aspect of the device. For this reason, the concept design shown in **Figure 1** has been designed to have a clear spatial delineation between the robotics while also keeping the aperture and range shifter assembly compact. We did consider adding distance between the aperture and range shifter. The results in Section 3.4 showed that if the distance between the range shifter and the aperture were increased, the beamlet would be further collimated (60), reducing the spot sizes further (34). However, the cost for adding separation is a significant reduction in the beamlet transmission, shown in **Figure 11**. With the range shifter placed against the aperture, the beamlet transmission is maximized, albeit while having a slightly larger spot size (61). The beamlet transmission reduces about 4 times faster than the spot sizes. We do not want to sacrifice the beamlet transmission further. For this reason, our work has mostly focused on abutting the range shifter and aperture. Only in scenarios where spot size must be reduced without regard to the beamlet transmission or where the field size is very small should increasing the distance between the aperture and range shifter of the SSA be considered.

Although there are some slight dosimetric advantages of using tungsten versus nickel due to its high density, including a slightly better beamlet transmission and higher peak dose to entrance dose ratio, the main difference in the aperture materials was found in their capacity to produce neutrons (62). This is a general concern for the SSA since the production of neutrons is relatively high compared to other aperture systems since beamlets are collimated spot by spot, unlike dynamic apertures or static apertures. The benefit of high lateral dose resolution offered by the SSA may outweigh the concern for secondary cancers caused by neutrons in many patients (63). Since the SSA concept must make a choice in aperture material, our focus was on the relative production of neutrons, dependent on treatment depth and material choice. This work does not attempt to quantify the neutron equivalent dose, but rather the physical dose. What we can see in **Figure 12** that the most important parameter is the maximum treatment depth. However, at any depth, nickel is preferable, resulting in about 60% of the neutron dose relative to using a tungsten aperture.

From a practical perspective (not considering the dosimetry), the main drawback of the SSA is the additional treatment time required for the device synchronization with the spot positions. Although the beam-on time must be increased, the main contributing factor to the added treatment time will likely be due to the time required for the SSA to move into position for each spot. The additional treatment delivery time is approximately equal to $t_{add}=N(\frac{x}{v})$ , where *N* is the number of spots, *x* is the spot spacing, and *v* is the motor speed of the SSA. For a 50 cm/s motor speed and 5 mm spot spacing, the additional time is 10 seconds for every 1000 spots utilizing the SSA. For a more precise estimate, the time added will also depend on the acceleration and deceleration of the device, which will require experimentation.

Building an analytical dose calculation model for the SSA would be challenging since the dose is sensitive to the precise geometry of the aperture and its position relative to the patient (and isocenter). In general, the spot shape cannot be modelled by a single Gaussian, multiple Gaussian (20), or combination of Gaussian plus Lorentz-Cauchy (64) and will vary in a non-trivial way with the proton energy. Likewise, the dosimetric characteristics along the depth dimension are highly dependent on the physics of interactions between the protons and the aperture material. For these reasons, the most reasonable approach towards clinical implementation is to incorporate the SSA into a Monte-Carlo-based treatment planning system. While the dosimetry is complex, likely requiring a Monte-Carlo dose engine, the SSA geometry is very simple to model – the cylindrical opening is fully described by the radius and thickness and the aperture opening is always aligned with the beamlet axis. This contrasts with MLC-like dynamic apertures/collimators, where the geometry is complex, and the number of free parameters is large. The only free parameter for calculating dose in the beam’s eye view coordinate with the SSA is the position in the spot direction (z-position) since the lateral position (x- and y-position) and the rotation of the SSA are determined based on the spot position. Because there is only one free parameter (z-position) for the SSA during spot delivery, it is natural to have the z-position of the SSA as a variable in the proton plan optimization. On the contrary, usually dynamic aperture parameters are considered retrospectively after the plan is optimized to get a better target dose penumbra. Including the aperture prospectively during the proton plan optimization is a new and exciting dimension of freedom in treatment planning that could lead to highly conformal treatments of shallow tumors.

The SSA has been shown to be able to greatly reduce the spot size as intended. The tradeoff for reduced spot sizes is excess integral neutron dose, poorer LET characteristics, and additional treatment time, all of which will require careful consideration in optimizing the SSA design. This simulation study has focused on proton dosimetry in water. As a pathway to clinical application, future work will include retrospective treatment planning and experimental validation of patient treatment plans to estimate the potential dosimetric benefits for patients. More practical concerns will need to be addressed as well, such as mechanical interference (collisions) between the aperture and the patient, an accurate prediction of treatment time, synchronization with spot positions, and engineering in general.

## Data availability statement

The raw data supporting the conclusions of this article will be made available by the authors, without undue reservation.

## Author contributions

JH formulated the concept, created the new modeling tool, wrote the manuscript. JS advised the concept, was a major editor of the manuscript. SP, WW, and RF (each are MDs) gave clinical motivation, edited/commented on the manuscript, generally advised medical side, MB advised the concept, was a major editor of the manuscript. WL supervised the project, established funding, advised the concept, helped formulate the modeling tool, major editor of the manuscript. All authors contributed to the article and approved the submitted version.

## Funding

This research was supported by Arizona Biomedical Research Commission Investigator Award, the Lawrence W. and Marilyn W. Matteson Fund for Cancer Research, and the Kemper Marley Foundation.

## Conflict of interest

The authors declare that the research was conducted in the absence of any commercial or financial relationships that could be construed as a potential conflict of interest. Dr. Jason Holmes and Dr. Wei Liu submitted a patent that covers the concept of spot scanning aperture on behalf of Mayo Clinic.

The remaining authors declare that the research was conducted in the absence of any commercial or financial relationships that could be construed as a potential conflict of interest.

## Publisher’s note

All claims expressed in this article are solely those of the authors and do not necessarily represent those of their affiliated organizations, or those of the publisher, the editors and the reviewers. Any product that may be evaluated in this article, or claim that may be made by its manufacturer, is not guaranteed or endorsed by the publisher.
